# Supplementary material for: Association between Drinking Patterns and Incident Hypertension in Southwest China
Source: Int J Environ Res Public Health. 2022 Mar 23;19(7):3801. doi: 10.3390/ijerph19073801 (PMC8997936; doi:10.3390/ijerph19073801)
Supplement: Supplementary file 1 [file ijerph-19-03801-s001.zip › ijerph-1548438-supplementary.pdf]

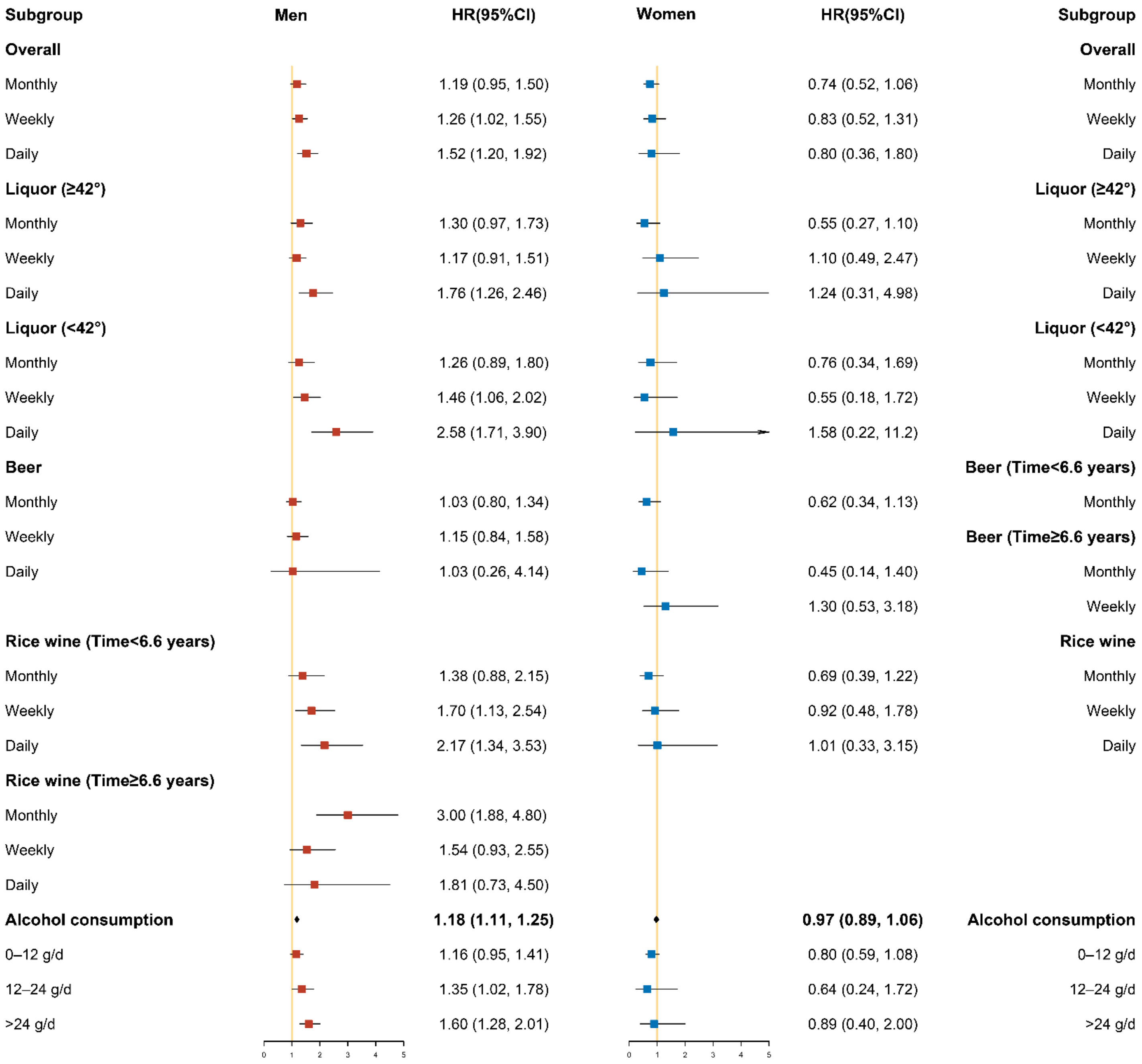


**Supplementary Figure S1. Subgroup analysis after stratification by gender (Model 1).**

Model 1 adjusts for age (continuous variable) and sex. Red squares represent the hazard ratio in men, blue squares represent the hazard ratio in women.

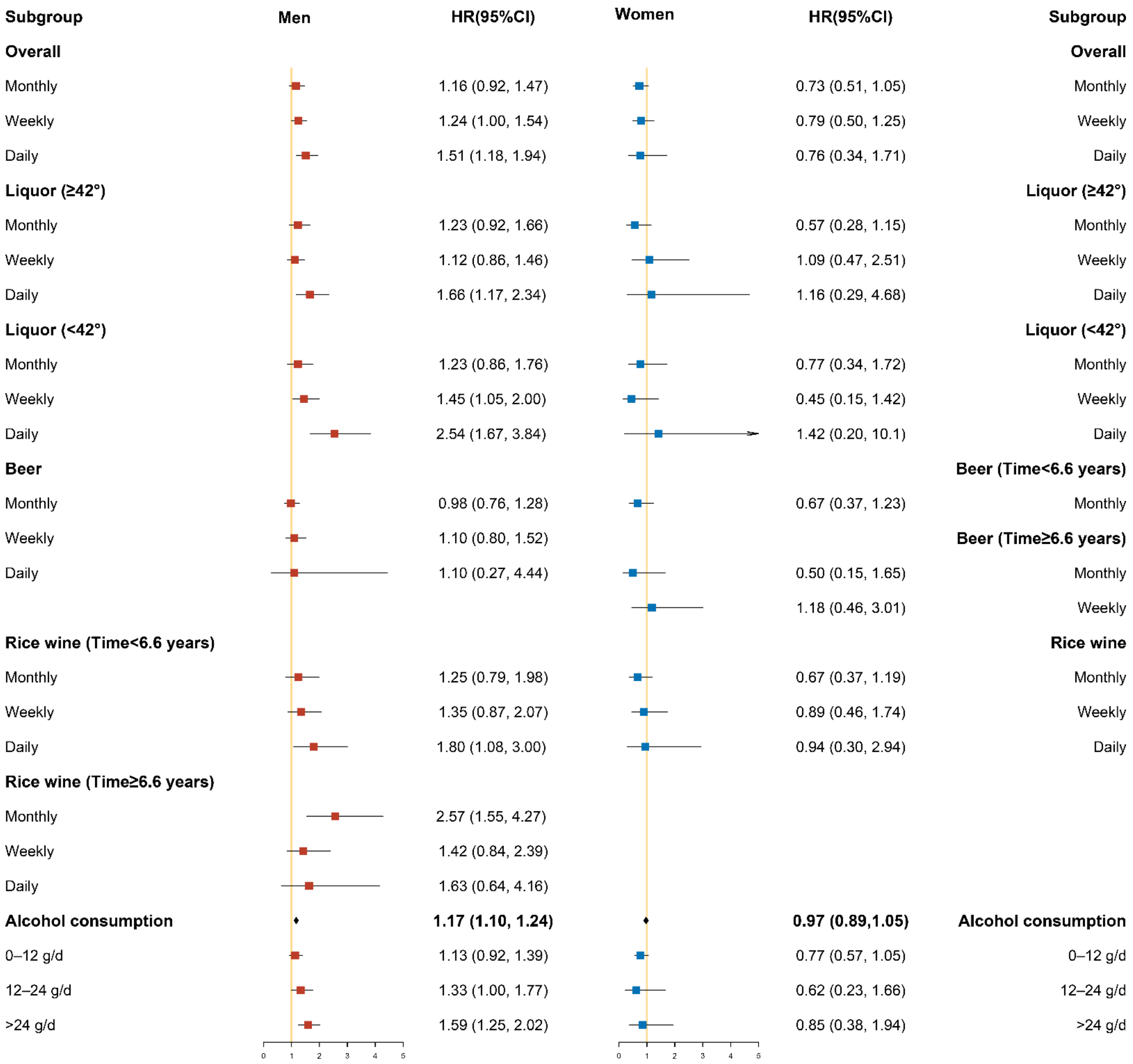


**Supplementary Figure S2. Subgroup analysis after stratification by gender (Model 2).**

Model 2 adjusts for age (continuous variable), sex, area, ethnicity, marriage, occupation, smoking status, exercise, and history of diabetes. Red squares represent the hazard ratio

in men, blue squares represent the hazard ratio in women.

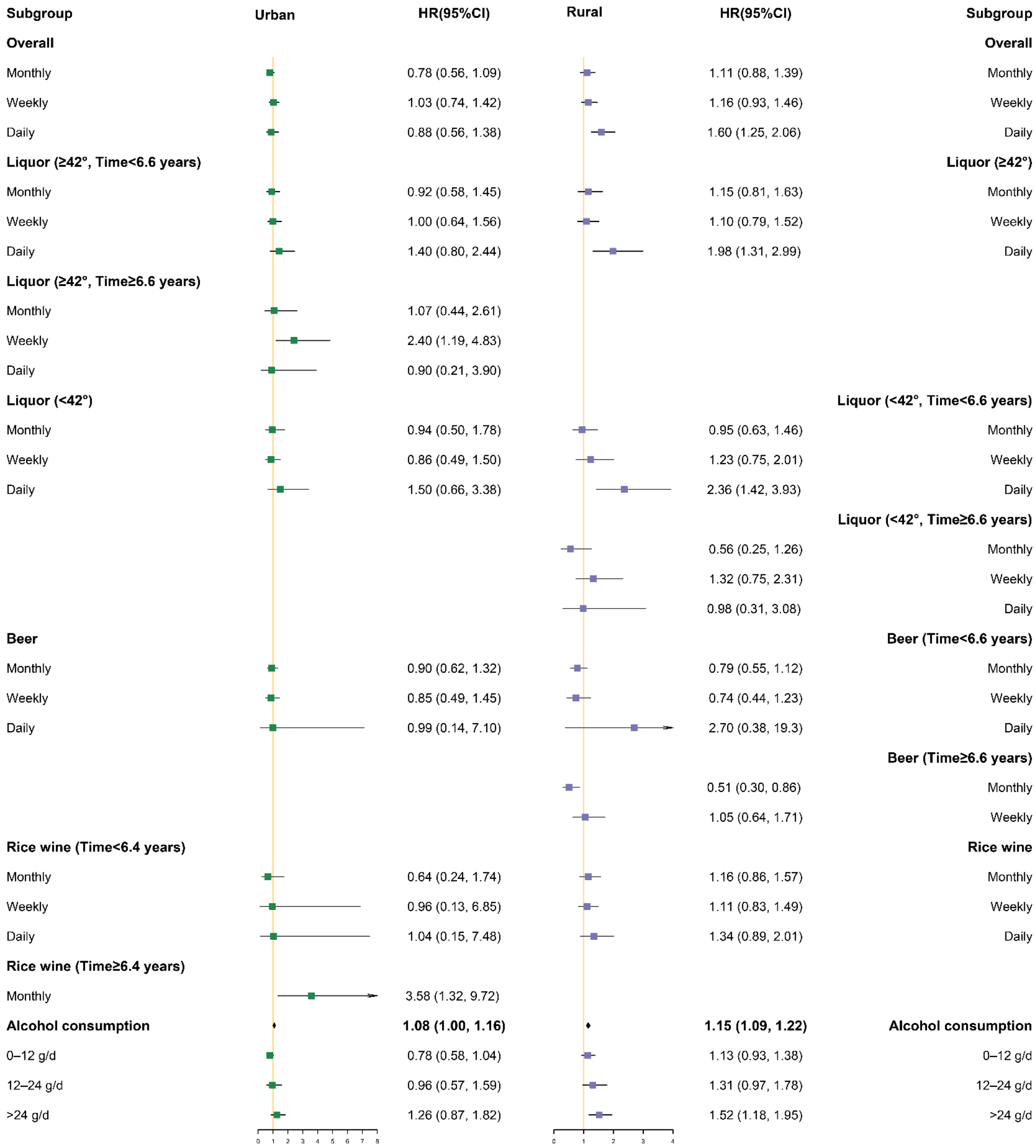


**Supplementary Figure S3. Subgroup analysis after stratification by urban and rural areas (Model 1).**

Model 1 adjusts for age (continuous variable), sex. Green squares represent the hazard ratio in urban areas, purple squares represent the hazard ratio in rural areas.

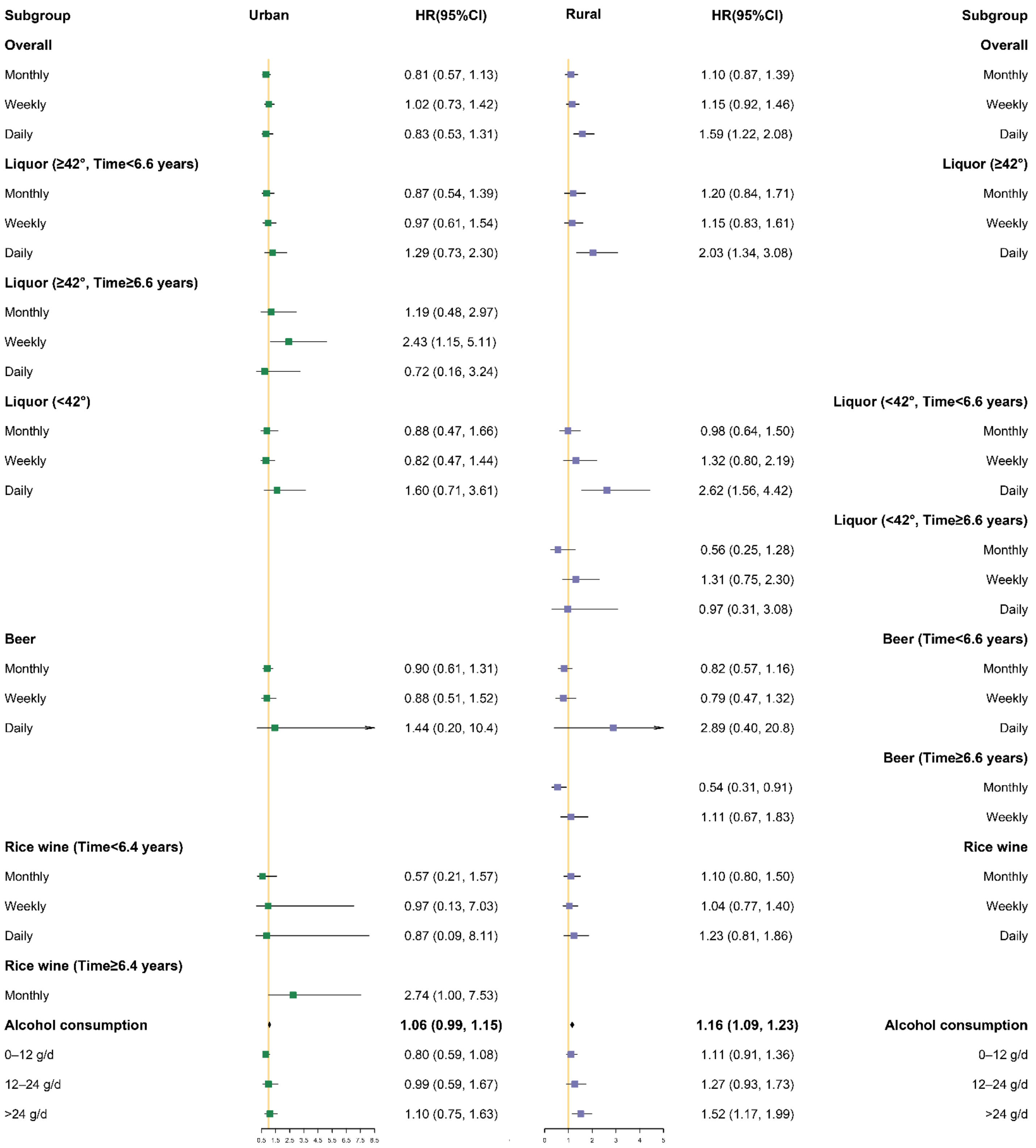


**Supplementary Figure S4. Subgroup analysis after stratification by urban and rural areas (Model 2).**

Model 2 adjusts for age (continuous variable), sex, area, ethnicity, marriage, occupation, smoking status, exercise, and history of diabetes. Green squares represent the hazard ratio in urban areas, purple squares represent the hazard ratio in rural areas.

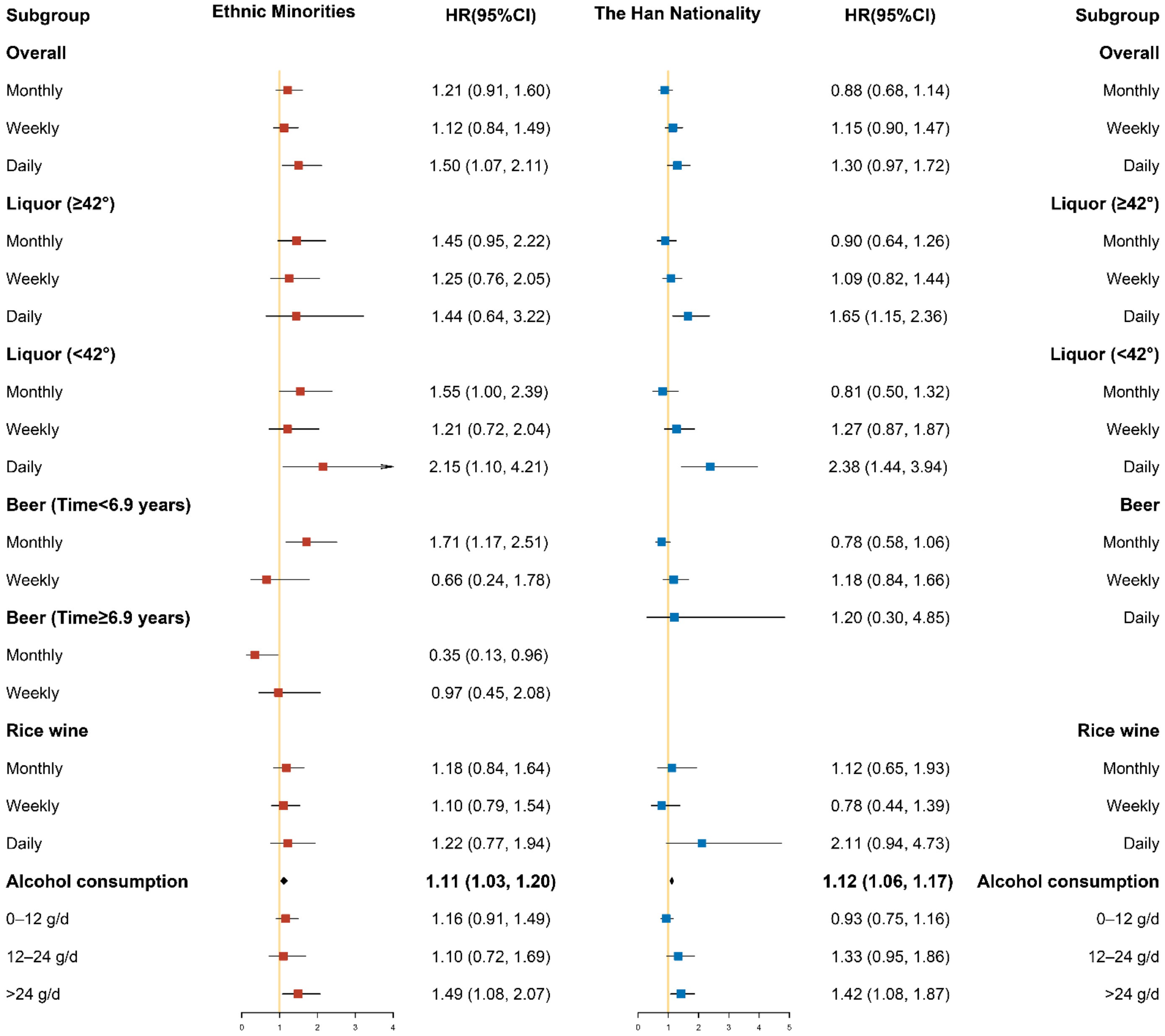


**Supplementary Figure S5. Subgroup analysis after stratification by ethnic minority and the Han nationality (Model 1).**

Model 1 adjusts for age (continuous variable) and sex. Red squares represent the hazard ratio in ethnic minorities, blue squares represent the hazard ratio in Han.

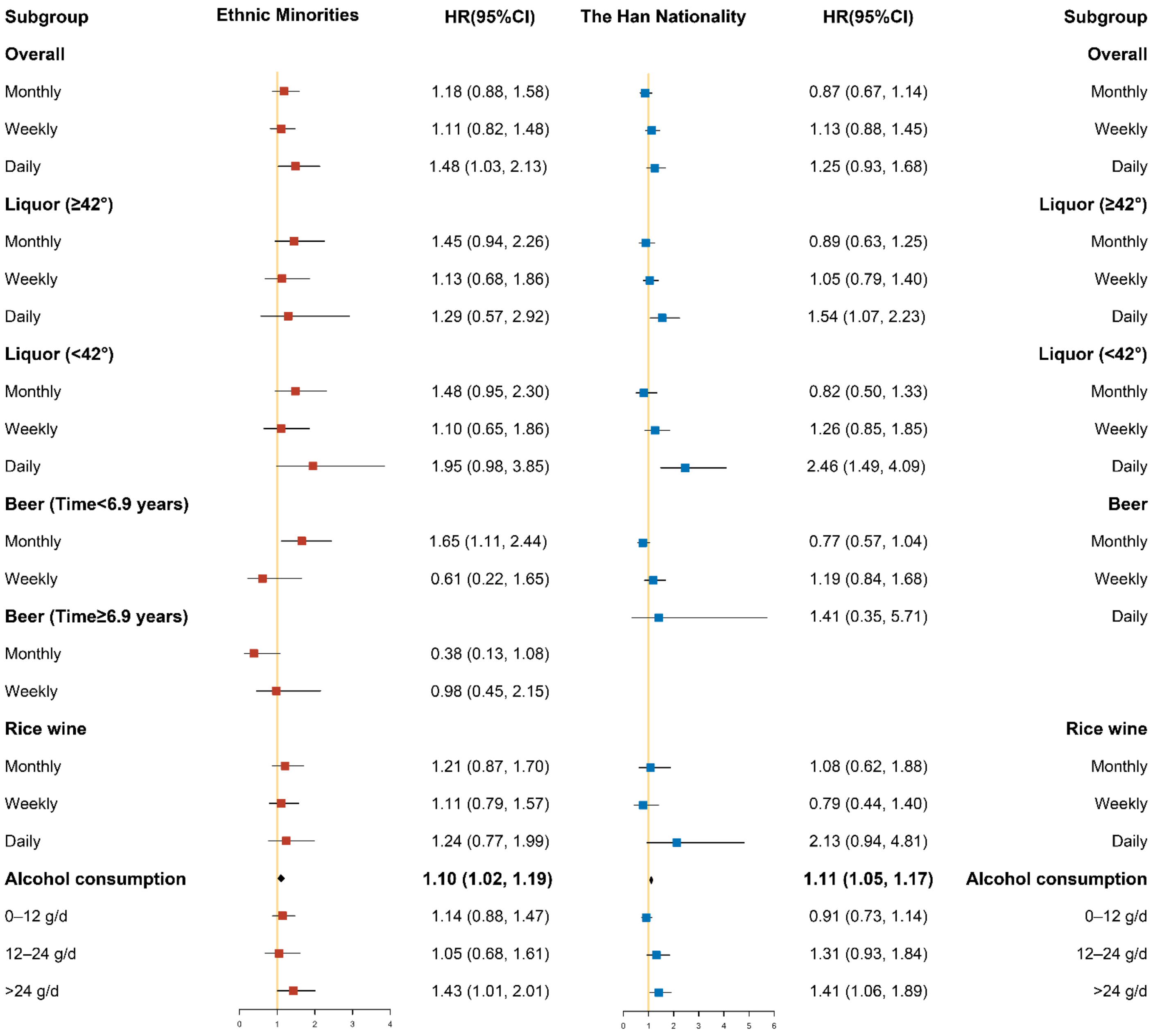


**Supplementary Figure S6. Subgroup analysis after stratification by ethnic minority and the Han nationality (Model 2).**

Model 2 adjusts for age (continuous variable), sex, area, ethnicity, marriage, occupation, smoking status, exercise, and history of diabetes. Red squares represent the hazard ratio in ethnic minorities, blue squares represent the hazard ratio in Han.

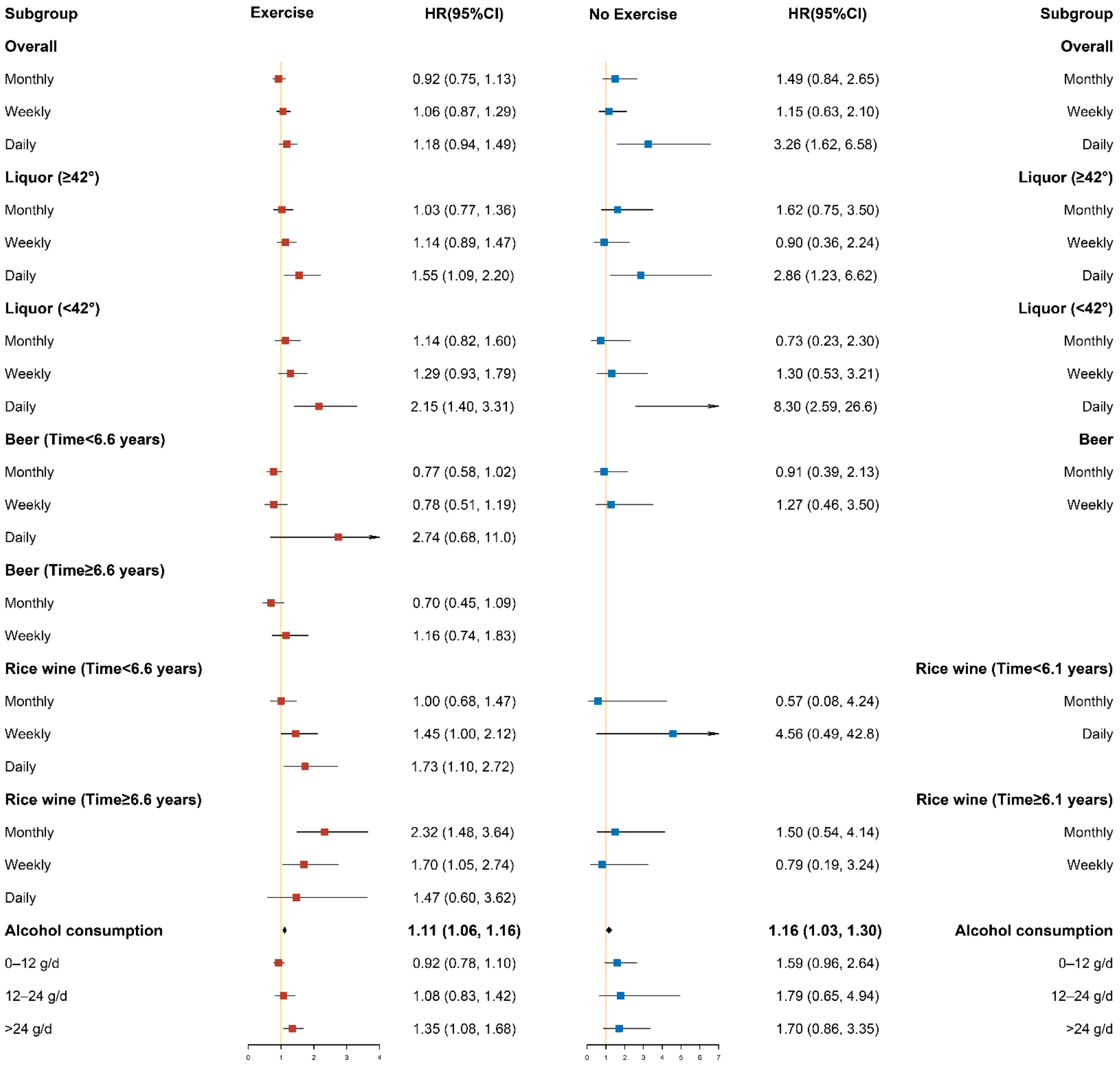


**Supplementary Figure S7. Subgroup analysis after stratification by exercise (Model 1).**

Model 1 adjusts for age (continuous variable) and sex. Red squares represent the hazard ratio in exercise population, blue squares represent the hazard ratio in no exercise

population.

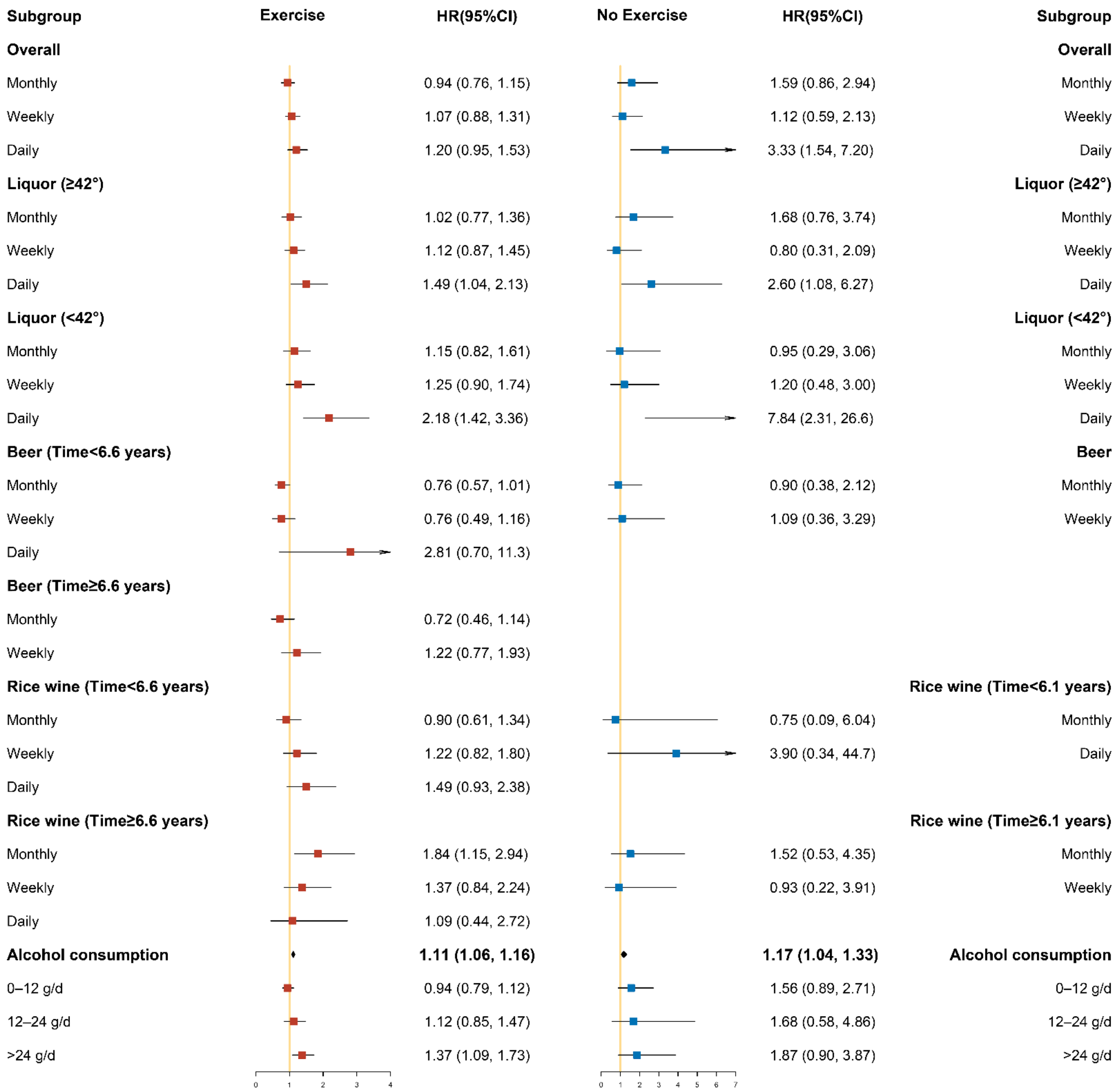


**Supplementary Figure S8. Subgroup analysis after stratification by exercise (Model 2).**

Model 2 adjusts for age (continuous variable), sex, area, ethnicity, marriage, occupation, smoking status, exercise, and history of diabetes. Red squares represent

the hazard ratio in exercise population, blue squares represent the hazard ratio in no exercise population.

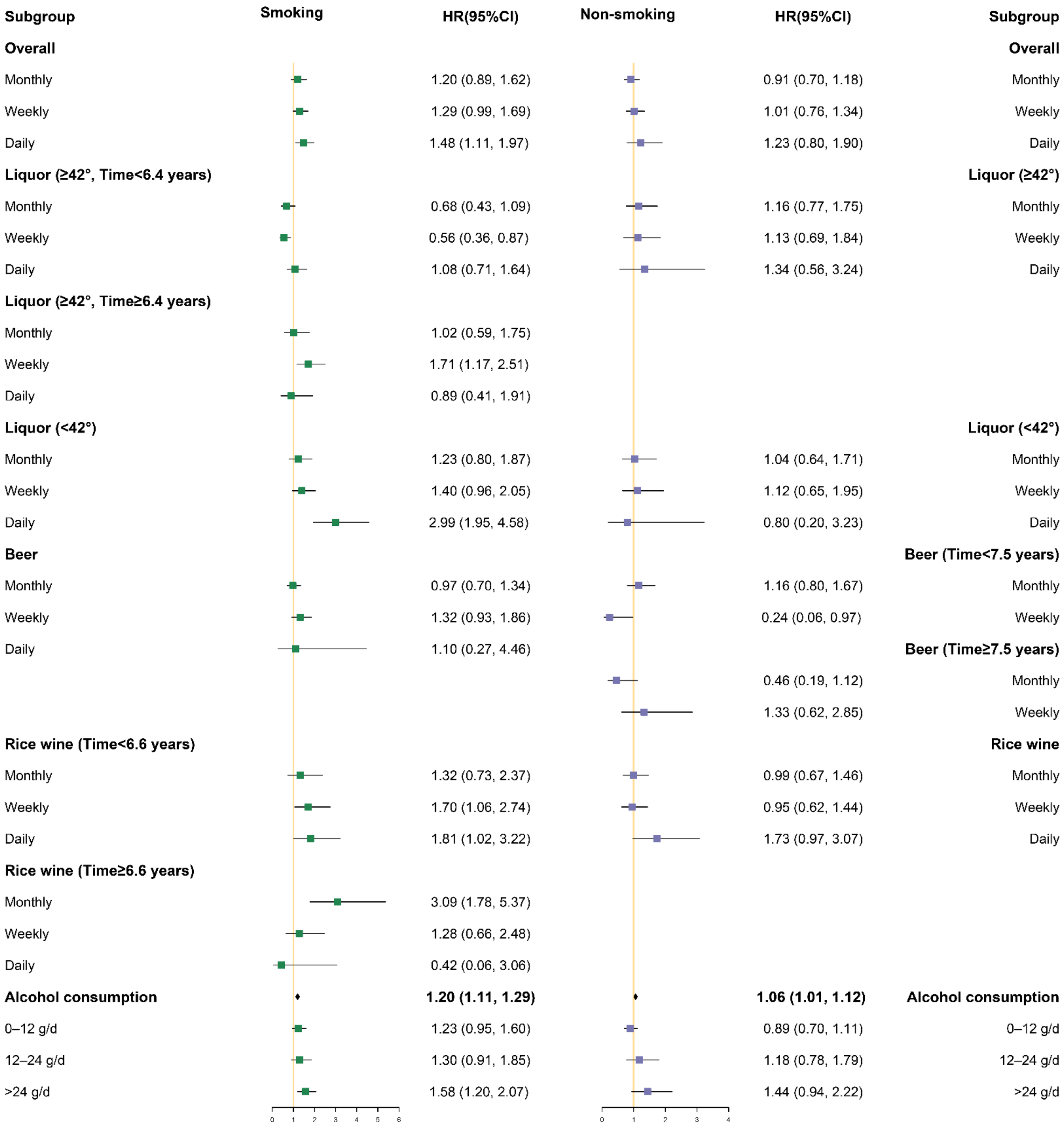


**Supplementary Figure S9. Subgroup analysis after stratification by smoking (Model 1).**

Model 1 adjusts for age (continuous variable) and sex. Green squares represent the hazard ratio in smokers, purple squares represent the hazard ratio in non-smokers.

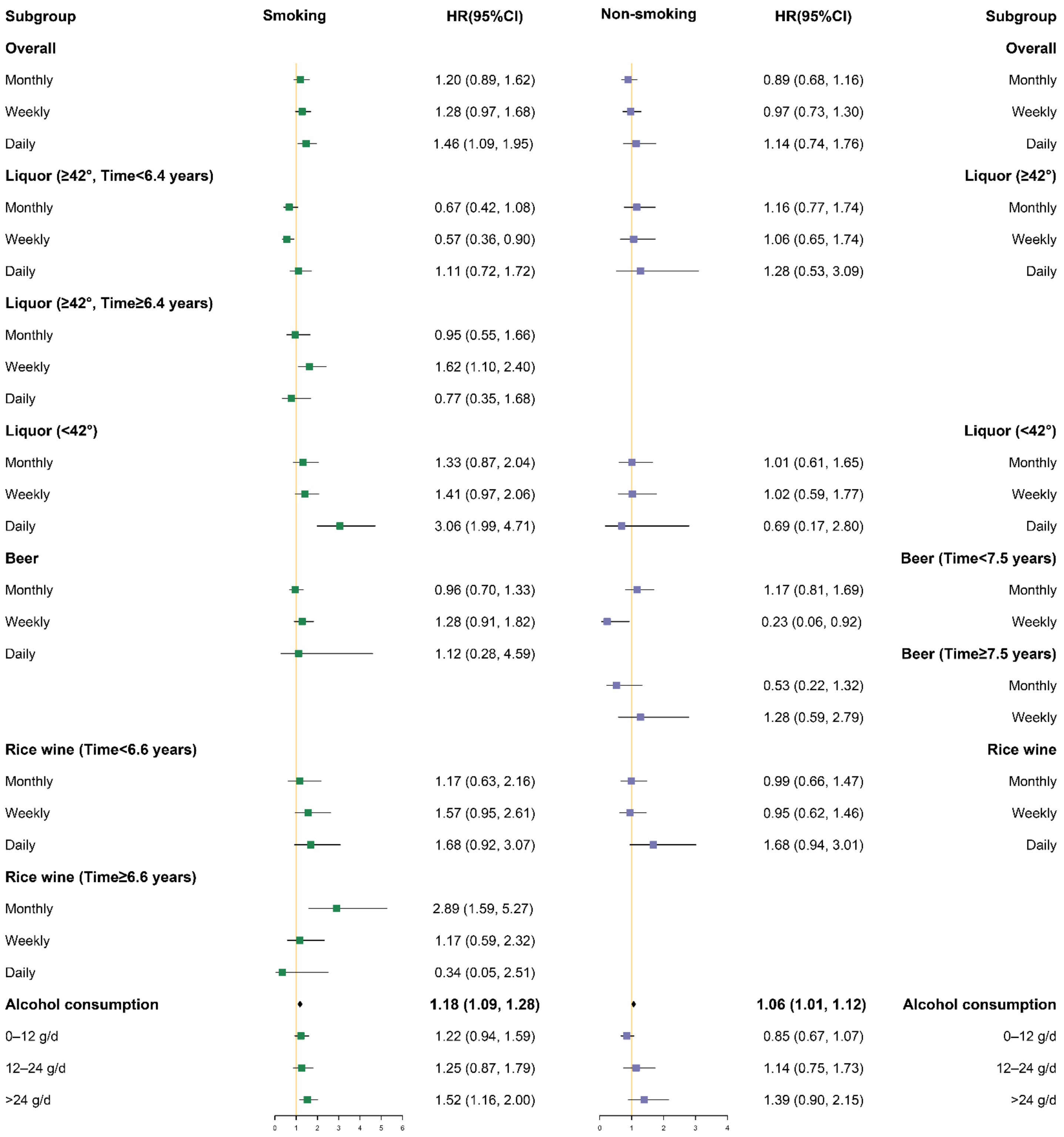


**Supplementary Figure S10. Subgroup analysis after stratification by smoking (Model 2).**

Model 2 adjusts for age (continuous variable), sex, area, ethnicity, marriage, occupation, smoking status, exercise, and history of diabetes. Green squares represent the hazard ratio in smokers, purple squares represent the hazard ratio in non-smokers.

**Supplementary Table S1. Hazard ratios (95% confidence intervals) of hypertension associated with the measured covariates.**

| Covariates | Level | HR (95%CI) | | |
|---|---|---|---|---|
| | | Model 1 | Model 2 | Model 3 |
| Age | Age | 1.03 (1.03, 1.04) | 1.03 (1.03, 1.04) | 1.03 (1.03, 1.04) |
| Sex | Men vs. Women | 1.11 (0.98, 1.26) | 1.19 (1.02, 1.39) | 1.14 (0.98, 1.33) |
| Area | Rural vs. Urban | 0.75 (0.67, 0.86) | 0.68 (0.59, 0.79) | 0.72 (0.62, 0.84) |
| Ethnicity | Yes vs. No | 0.98 (0.87, 1.10) | 1.07 (0.94, 1.21) | 1.15 (1.01, 1.31) |
| Marriage | Unmarried vs. Married | 0.86 (0.68, 1.11) | 0.88 (0.69, 1.14) | 0.91 (0.70, 1.17) |
| | Others vs. Married | 1.01 (0.84, 1.22) | 1.03 (0.85, 1.24) | 1.02 (0.85, 1.24) |
| Occupation | Others vs. Farmer | 0.86 (0.75, 0.99) | 0.79 (0.68, 0.91) | 0.80 (0.69, 0.93) |
| | Unemployed or retired vs. Farmer | 0.86 (0.73, 1.02) | 0.82 (0.68, 0.98) | 0.81 (0.67, 0.96) |
| Smoking status | Yes vs. No | 0.97 (0.82, 1.15) | 0.92 (0.78, 1.09) | 0.94 (0.79, 1.11) |
| Exercise | Yes vs. No | 1.35 (1.14, 1.60) | 1.23 (1.03, 1.47) | 1.12 (0.93, 1.34) |
| History of diabetes | Yes vs. No | 0.99 (0.80, 1.24) | 1.01 (0.81, 1.27) | 0.94 (0.75, 1.18) |
| BMI | BMI | 1.04 (1.03, 1.06) | 1.04 (1.02, 1.06) | 1.03 (1.01, 1.05) |
| SBP | SBP | 1.01 (1.01, 1.02) | 1.01 (1.01, 1.02) | 1.01 (1.01, 1.02) |
| Triglycerides | Triglycerides | 1.02 (0.99, 1.06) | 1.02 (0.99, 1.05) | 1.05 (1.01, 1.08) |
| Total cholesterol | Total cholesterol | 0.97 (0.93, 1.02) | 0.97 (0.92, 1.01) | 0.84 (0.78, 0.91) |
| HDL cholesterol | HDL cholesterol | 0.94 (0.86, 1.02) | 0.90 (0.82, 0.99) | 1.06 (0.95, 1.19) |
| LDL cholesterol | LDL cholesterol | 1.07 (1.02, 1.13) | 1.06 (1.01, 1.11) | 1.18 (1.10, 1.27) |

Note: Model 1: adjusted for age (continuous variable), sex, and HRs (95%CIs) for age

and sex are in original model;

Model 2: Model 1 plus area, ethnicity, marriage, occupation, smoking status, exercise, and history of diabetes, and HRs (95%CIs) for age, sex, area, ethnicity, marriage, occupation, smoking status, exercise, and history of diabetes are in original model;

Model 3: Model 2 plus SBP, total cholesterol, triglycerides, HDL-C value, LDL-C value, baseline BMI value.

Abbreviations: HR, hazard ratio; 95%CI, 95% confidence interval.
